# Antibiotic-Driven Gut Microbiome Disorder Alters the Effects of Sinomenine on Morphine-Dependent Zebrafish

**DOI:** 10.3389/fmicb.2020.00946

**Published:** 2020-05-15

**Authors:** Zhu Chen, Chen Zhijie, Zhou Yuting, Xiao Shilin, Zhou Qichun, Ou Jinying, Luo Chaohua, Li Jing, Mo Zhixian

**Affiliations:** ^1^School of Traditional Chinese Medicine, Southern Medical University, Guangzhou, China; ^2^Central Laboratory, Southern Medical University, Guangzhou, China

**Keywords:** morphine, sinomenine, microbiota-gut-brain axis, zebrafish, CPP

## Abstract

Morphine is one of the most severely abused drugs in the world. Previous research on morphine addiction has focused on the central nervous system (CNS). Studies have shown that a two-way regulation of the brain and gut microbiota (GM), suggesting a link between GM and CNS disease. However, the functional mechanism underlying the relationship between intestinal flora and morphine dependence is unclear. In this study, the effect of sinomenine on morphine addiction was evaluated based on the microbiota-gut-brain axis (MGBA). The results show that the GM plays an important role in morphine dependence. Morphine treatment induced zebrafish conditional position preference (CPP), and significantly changed zebrafish GM characteristics and the expression of MGBA-related genes in the zebrafish brain and intestine. Importantly, sinomenine, an alkaloid with a similar structure to morphine, can reverse these morphine-induced changes. Subsequently, morphine-dependent CPP training was performed after antibiotic administration. After antibiotic treatment, zebrafish CPP behavior, the composition and proportions of the zebrafish GM, and the expression of MGBA-related genes in zebrafish were changed. More interestingly, sinomenine was no longer effective in treating morphine dependence, indicating that antibiotic-driven intestinal flora imbalance alters the efficacy of sinomenine on morphine-dependent zebrafish. This study confirms that the MGBA is bidirectionally regulated, highlighting the key role of the GM in the formation and treatment of morphine dependence, and may provide new treatment strategies for using traditional Chinese medicine to treat drug addiction.

## Introduction

Morphine is an opioid receptor agonist with excellent analgesic effects. In clinical practice, morphine is often used for surgery, trauma, severe pain accompanying burns, or three-step analgesia in patients with advanced cancer. However, morphine tolerance and addiction limit its clinical application. Morphine addiction not only harms the central nervous system (CNS) of the body but also affects the peripheral system. Studies have found that adverse reactions in the gastrointestinal system of morphine users are common (Baldini et al., 2012).

With the deepening of “microbiota-gut-brain axis” (MGBA) research in recent years, the relationship between the brain, gut, and intestinal microbiota has been gradually revealed. Numerous studies have shown that CNS disease is closely related to the intestinal microbiota. The intestinal microbiota can affect CNS function, behavior, and cognition through endocrine, neural, immune, and metabolic pathways; in turn, the brain can regulate these four signaling pathways to affect the composition and function of the intestinal microbiota. Compared with healthy people, the intestinal flora of patients with autism shows significantly changes (Finegold et al., 2010; Adams et al., 2011; Kang et al., 2013). Symbiotic microorganisms in the host affect a variety of complex behaviors, including social, emotional, and anxiety-like behaviors, and are also involved in mouse and human brain function and development (Collins et al., 2012; Tillisch et al., 2013). Studies in germ free (GF) animals and in animals exposed to pathogenic bacterial infections, probiotics, or antibiotic drugs have shown that gut microbes play a role in regulating anxiety, mood, cognition, and pain (Cryan and Dinan, 2012). The emerging concept of the MGBA shows that mutual regulation of the gut microbiota (GM) and the brain may be a new approach to treating complex CNS diseases.

Sinomenine is the main active ingredient of the traditional Chinese medicine *Sinomeniumacutum*, which has analgesic, anti-inflammatory, and immunoregulatory effects (Zhu et al., 2017b). Sinomenine is a morphine-related alkane alkaloid, whose structure is similar to morphine but which has no addictive properties (Ou et al., 2018). Our previous results showed that sinomenine can inhibit the withdrawal contraction response caused by morphine-dependence and the withdrawal response induced by naloxone in morphine-dependent mice; furthermore, sinomenine can resist the conditional position preference (CPP) effect and the increase of histamine and cAMP levels in the morphine-dependent mouse brain (Wang et al., 2003; Mo et al., 2006).

Based on the MGBA, this study aimed to establish an in vivo morphine-dependent zebrafish animal model under conditions of a normal or a disordered GM, to analyze the characteristics of the intestinal flora in morphine-dependent zebrafish. Furthermore, it aimed to study the interaction between the microbiota and morphine dependence, and to study the effect of sinomenine on morphine dependence.

## Materials and Methods

### Animals

Wild-type adult male zebrafish (AB strain, age: 3–6 months old; weight: 0.3–0.5 g) were provided by Laboratory of Zebrafish Modeling and Drug Screening for Human Diseases (Guangzhou, China). All animals were maintained in a multi-tank system (temperature: 28°C; 14 h light: 10 h dark cycle) to simulate their environmental condition. Adult zebrafish were fed twice a day with a mixture of flake fish food and live brine shrimps.

### Conditioned Position Preference Test

**FIGURE 1:**
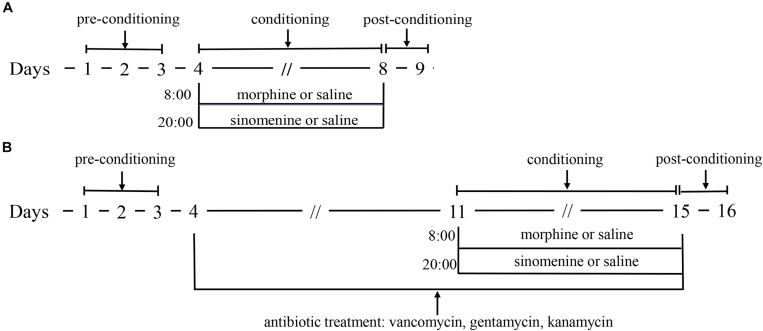
**(A)** is the schematic of morphine dependent zebrafish CPP experiment; **(B)** is the schematic of morphine dependent zebrafish CPP experiment with antibiotic treatment.

As described in a previous study (Zhu et al., 2017a), the conditioned position preference (CPP) apparatus consisted of a black box and a white box, with the CPP test divided into three phases. The first phase was the pre-conditioning phase (days 1–3), during which the zebrafish were fed in the CPP apparatus for 15 min per day. On day 3, the residence time of zebrafish in the CPP box during a 15 min period was recorded, and zebrafish with natural preference for the white compartment were excluded. The remaining zebrafish were randomly divided among three groups: (1) the control group (C); (2) the morphine group (M); and the morphine+sinomenine group (M+S). During the conditioning phase (days 4–8), on days 4, 6, and 8 at 08:00, zebrafish from the M and M+S groups were injected with morphine (40 mg/kg), while zebrafish from C group were injected with an equal volume of saline. The zebrafish were immediately placed in the white compartment (drug-pair box) for 45 min. On days 5 and 7 at 08:00, the zebrafish from all groups were injected with saline, and then immediately placed in the black compartment for 45 min. At 20:00 every day, zebrafish from the C and M groups received a saline injection, whereas M+S group zebrafish were injected with a sinomenine solution (80 mg/kg). In phase 3 (day 9), 24 h after the last injection of morphine, the CPP test was performed to record the residence time of zebrafish in the white and black compartments (Figure 1A).

The administration of antibiotics to test animals is a commonly used method to study the effect of GM. Compound antibiotics consisting of vancomycin, gentamicin, and kanamycin have good antibacterial effect on Gram-positive and Gram-negative bacteria and these antibiotics are oral antibiotics that that are not absorbed by the intestine. The antibiotic intervention morphine-dependent CPP experiment was performed similarly to the CPP test. After the first stage, the zebrafish were randomly divided into three groups: (1) the antibiotic group (A); (2) the antibiotic+morphine group (A+M); and (3) the antibiotic+morphine+sinomenine group (A+M+S). All zebrafish were fed an antibiotic solution (vancomycin, 100 mg/L; gentamicin, 10 mg/L, and kanamycin, 5 mg/L). After this procedure, all of the experimental protocols for the conditioning and post-conditioning phases were the same as those for the CPP test (Figure 1B).

### 16S rRNA Gene Sequencing

After the CPP test, the zebrafish were sacrificed by freezing and their intestinal contents were removed under aseptic conditions. Microbial DNA was extracted using E.Z.N.A.^®^ soil DNA Kit (Omega Bio-tek, Norcross, GA, United States), and the concentration and purity were measured using the NanoDrop One (Thermo Fisher Scientific, MA, United States). 16S rRNA genes from V3–V4 regions were amplified using the specific primers 338F (5′-ACTCCTACGGGAGGCAGCAG-3′) and 806R (5′-GGACTACHVGGGTWTCTAAT-3′) on a Bio-Rad S1000 Thermal Cycler (Bio-Rad Laboratories, CA, United States). The length and concentration of the PCR product were detected by 1% agarose gel electrophoresis. Sequencing libraries were generated using a NEBNext^®^ Ultra^TM^ II DNA Library Prep Kit for Illumina^®^ (New England Biolabs, MA, United States) following the manufacturer’s recommendations, and index codes were added. Library quality was assessed on a Qubit^®^ 2.0 Fluorometer (Thermo Fisher Scientific, MA, United States). Finally, the library was sequenced on an Illumina Nova6000 platform and 250 bp paired-end reads were generated (Guangdong Magigene Biotechnology Co., Ltd., Guangzhou, China). Fastp (version 0.14.1^1^) was used to control the quality of the raw data and cutadapt^2^ was used to obtain the paired-end clean reads. Operational taxonomic units (OTUs) were clustered with a 97% similarity cutoff using UPARSE. For each representative sequence, the SILVA database^3^ was used to annotate taxonomic information using the command “usearch-sintax” (setting the confidence threshold to the default of ≥0.8).

### qPCR Assay

Total RNA was extracted from zebrafish using the TRIzol extract reagent (Takara, Dalian, China), as previously described (Fang et al., 2017), and reversed-transcribed into cDNA using a PrimerScript RT Reagent Kit (Takara, Dalian, China). qPCR was performed on a Light Cycler 96 system (Roche, Germany) using the TB Green Premix Ex Taq Reagent Kit (Takara, Dalian, China), according to the manufacturer’s recommendation. The primers used in this experiment were designed by RiboBio Co., Ltd., (Guangzhou, China) and are listed in Table 1.

**TABLE 1:** PCR primer sequences for mRNAs.

mRNA	PCR primer (5′–3′)
zf-b-actin F	ATGGATGAGGAAATCGCTG
zf-b-actin R	ATGCCAACCATCACTCCCTG
zf-il1b F	TGGACTTCGCAGCACAAAATG
zf-il1b R	GTTCACTTCACGCTCTTGGATG
zf-oprm1 F	ACGAGCTGTGCAAGATTGTG
zf-oprm1 R	CCGATTGCAGATGAAAGGAT
zf-oprd1 F	ACTATGAGAGCGTGGACCGTT
zf-oprd1 R	GCGGAGGAGAGGATCCAGAT
zf-htr2aa F	GCATCTCTCTTACCCAATCTATCC
zf-htr2aa R	AACTAACTCCTCTTTGGTCGTCTC
zf-drd2a F	TGGTACTCCGGAAAAGACG
zf-drd2a R	ATCGGGATGGGTGCATTTC
zf-bdnf F	AACATTCCGTTTACATTCTC
zf-bdnf R	ACAACAGCACCTTGACATAG
zf-ntrk2 F	GGAAAAGCAAAAACCCTGTCTAGA
zf-ntrk2 R	TGTAGCATCACTTCCTGCCATT
zf-occludin a F	CTTCAGTGAGTTTCCTCCTATTGTG
zf-occludin a R	CCTGGTGGTCTTGATCAAAGAG
zf-occludin b F	TCTCCTTGTGTTTGCGGTGA
zf-occludin b R	GCCATCCTTAATGGTCTTAACGT

### Statistical Analysis

Values are expressed as mean ± SD. All data were analyzed using one-way analysis of variance (ANOVA), followed by the least significant difference (LSD) *post hoc* test (two-tailed). All statistical analyses were performed using SPSS software (version19.0). *P* < 0.05 was considered to be statistically significant.

## Results

### Sinomenine Inhibited Morphine-Dependent CPP in Zebrafish

**FIGURE 2:**
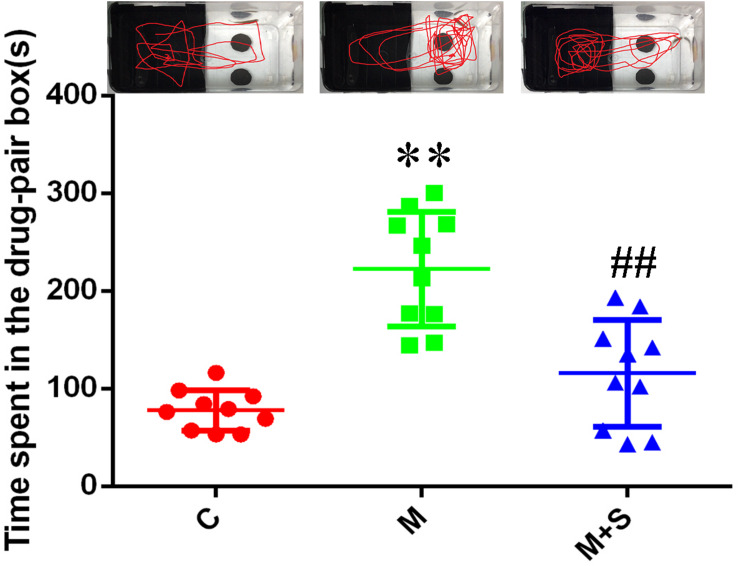
Time spent by zebrafish in the drug-pair compartment. (*n* = 8). ^∗∗^*P* < 0.01 vs the C group; ^##^*P* < 0.01 vs the M group. C, control group; M, morphine group; M+S, morphine+sinomenine group.

As shown in Figure 2, compared with the C group, the residence time in the drug-pair box for the M group zebrafish was significantly increased (*P* < 0.01). Furthermore, compared with M group, the residence time in the drug-pair box for the M+S group zebrafish was significantly decreased (*P* < 0.01).

### Sinomenine Inhibited Morphine-Induced Gut Microbiome Alterations in Zebrafish

**FIGURE 3:**
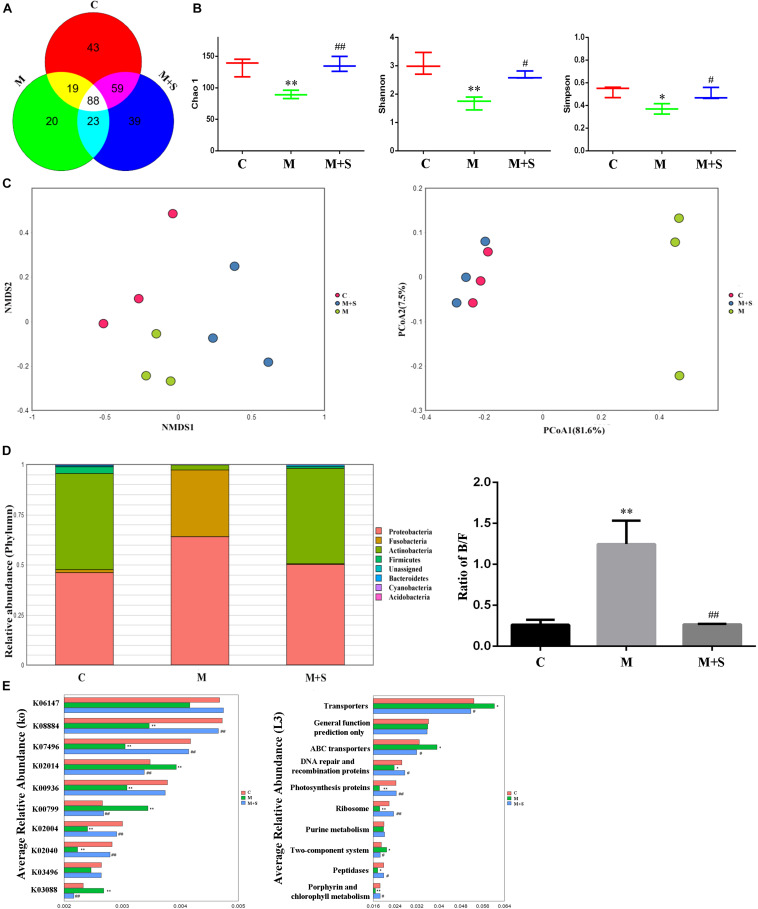
Effect of sinomenine use on the gut microbiome accompanying morphine dependence. **(A)** Venn diagram depicting the distribution of bacterial taxa (at the OTU level) from the different zebrafish experimental groups. **(B)** Alpha diversity of the fecal microbiome among the three groups according to the Chao 1, Richness, Shannon, and Simpson indices. **(C)** Beta diversity of the fecal microbiome among the three groups based on principal coordinate analysis (PCoA) and nonmetric multidimensional scaling (NMDS) at the OTU level based on Bray–Curtis similarity algorithms. **(D)** Comparison of species composition among the fecal microbiota of the C, M, and M+S groups at the phylum level. **(E)** Prediction of changed KEGG pathways using PICRUST analysis. *P < 0.05, ^∗∗^*P* < 0.01 vs the C group; ^#^*P* < 0.05, ^##^*P* < 0.01 vs the M group. C, control group; M, morphine group; M+S, morphine+sinomenine group.

Operational taxonomic unit is one of the most common terms in microbiology, which is man-made uniform flags for a taxonomic unit (strain, genus, species, grouping, etc.). As shown by the Venn diagram (Figure 3A), a total of 291 OTUs were identified among all the samples, 88 of which were shared by the three groups. A total of 43 OTUs were unique to the C group, 20 to the M group and 39 to the M+S group.

Alpha diversity analysis is often used to analyze the community richness and diversity. Chao1 index can reflect the community richness and Shannon index, Simpson index can reflect the community diversity. The community richness and diversity of the M group were significantly decreased, as indicated by the reduced Chao 1, Shannon, and Simpson index values (Figure 3B). Distance matrices (beta diversity) among samples were generated based on Bray–Curtis similarity algorithms at the OTU level, and are reported according to principal coordinate analysis (PCoA) and nonmetric multidimensional scaling (NMDS) (Figure 3C). The PCoA and NMDS results show differences in microbial profiles among the three groups (C, M, and M+S), with the distance between the C group and the M+S group being relatively small.

Based on the results of the analysis of GM characteristics, we continued our investigation by studying the composition of the zebrafish population at the GM phylum level. As shown in the Figure 3D, the zebrafish gut microbial is mainly divided into seven phyla: Proteobacteria, Fusobacteria, Firmicutes, Bacteroidetes, Actinobacteria, Cyanobacteria, and Acidobacteria. Compared with the C group, the proportion of Fusobacteria in the zebrafish intestinal microbiota of the M group was significantly increased; this was accompanied by a significant decrease in the proportion of Actinobacteria. The Bacteroidetes/Firmicutes (B/F) ratio of the M group was significantly up-regulated compared with the C group. More importantly, the above changes could be diminished by administering sinomenine.

The KEGG database is an important functional database that is used to annotate genes (Kanehisa et al., 2004). Genes can be projected into the KEGG PATHWAY database to reveal interactions with other genes that may influence the health of the host (Altermann and Klaenhammer, 2005). To correlate changes in intestinal microbiota with function, we predicted the KEGG pathway using a PICRUSt analysis of the zebrafish intestinal microbiota. As shown in Figure 3E, morphine treatment significantly decreased expression of the following genes: serine/threonine protein kinase (K08884); putative transposase (K07496); two-component system (K00936); putative ABC transport system permease protein (K02004); and phosphate transport system substrate-binding protein (K02040). In contrast, it increased the expression of genes encoding: iron complex outer-membrane receptor protein (K02014); glutathione S-transferase (K00799); and RNA polymerase (K03088). At level 3 in the KEGG analysis, the abundances of the following metabolic pathways were significantly altered: transporter; ABC transporters; DNA repair and recombination proteins; photosynthesis proteins; ribosome; two-component system; peptidases and porphyrin; and chlorophyll metabolism. More importantly, sinomenine treatment effectively inhibited the changes to the abundances of KEGG pathways in the zebrafish intestinal microbiota caused by morphine dependence.

### Sinomenine Inhibited Morphine-Induced MGBA-Relative Gene Alterations in Zebrafish

**FIGURE 4:**
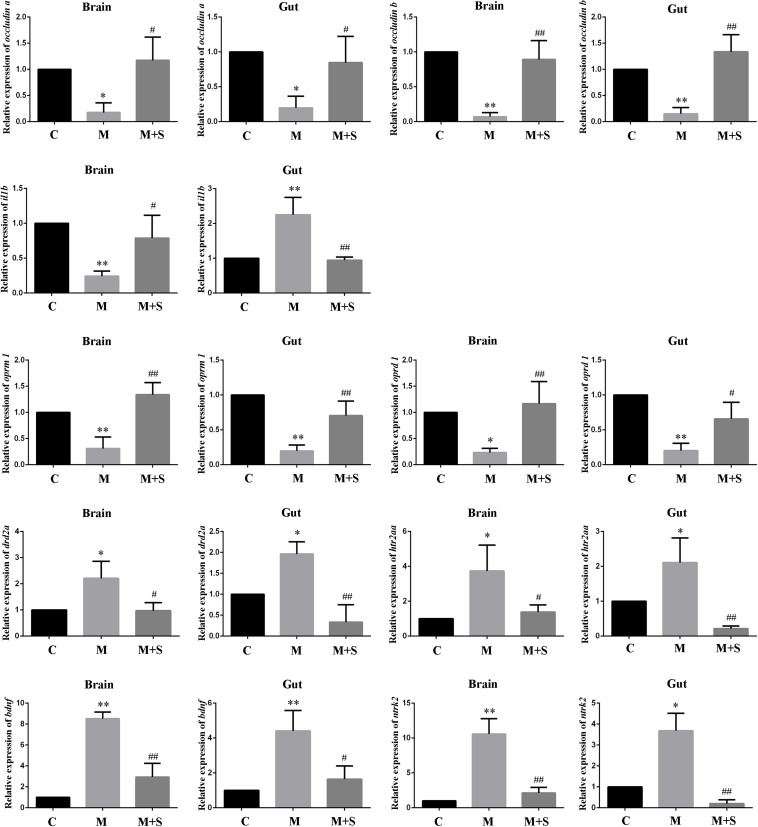
qPCR analysis of *occludin a*, *occludin b*, *il1b*, *oprm1*, *oprd1*, *drd2a*, *htr2aa*, *bdnf*, and *ntrk2* expression in zebrafish. (*n* = 3). ^∗^*P* < 0.05, ^∗∗^*P* < 0.01 vs the C group; ^#^*P* < 0.05, ^##^*P* < 0.01 vs the M group. C, control group; M, morphine group; M+S, morphine+sinomenine group.

As shown in Figure 4, compared with the C group, the expression levels of the tight junction proteins (*occludin a* and *occludin b*) in the zebrafish brain and intestine were significantly reduced after administration of morphine, while sinomenine treatment was able to return these expression levels to normal.

Compared with the C group, expression level of the *il1b* mRNA in the zebrafish brain was significantly lower after morphine treatment, whereas expression level in the intestinal tract of the zebrafish was significantly higher. The sinomenine intervention inhibited the *il1b* gene expression changes caused by morphine dependence both in the zebrafish brain and in the intestine.

Compared with the C group, the expression levels of *oprm1* and *oprd1* mRNAs in the M group were significantly reduced. Moreover, compared with the M group, the expression levels of *oprm1* and *oprd1* mRNAs in the M+S group zebrafish were significantly increased.

Compared with the C group, the expression levels of *drd2a* and *htr2aa* mRNAs in the M group zebrafish were significantly up-regulated. Furthermore, compared with the M group, the expression levels of *drd2a* and *htr2aa* mRNAs in the M+S group zebrafish were significantly down-regulated.

Compared with C group, the expression levels of the mRNAs of *bdnf* and its receptor *ntrk2* in the zebrafish were significantly increased after morphine treatment, while the sinomenine intervention significantly reduced the expression of these mRNA.

### Antibiotic Use Leads to the Inability of Sinomenine to Inhibit Morphine-Dependent CPP in Zebrafish

**FIGURE 5:**
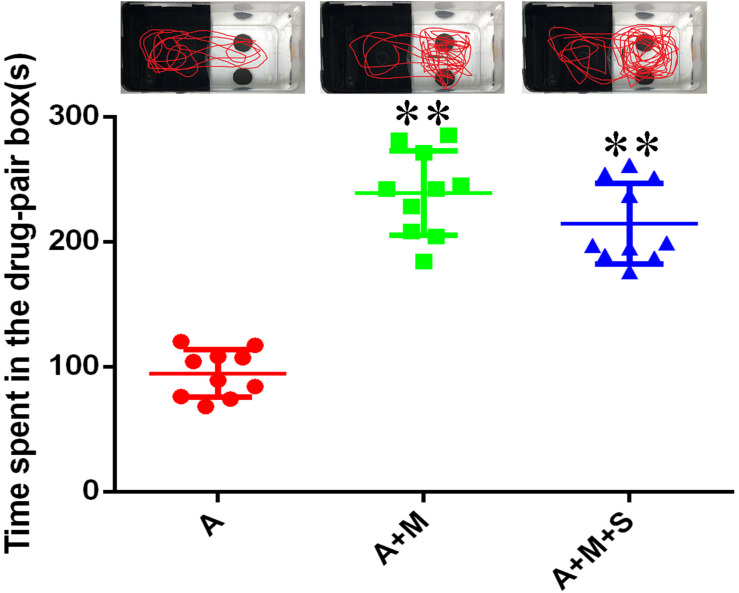
Time spent by zebrafish in the drug-pair compartment. (*n* = 8). ^∗∗^*P* < 0.01 vs the A group. A, antibiotic group; A+M, antibiotic+morphine group; A+M+S, antibiotic+morphine+sinomenine group.

As shown in Figure 5, compared with the A group, the residence times in the drug-pair box for the A+M and A+M+S group zebrafish were significantly increased (*P* < 0.01). Compared with the A+M group, there was no significant change of the residence time in the drug-pair box for the A+M+S group (*P* > 0.05).

### Antibiotic Use Attenuates the Effects of Sinomenine on Morphine-Induced Gut Microbiome Alterations in Zebrafish

**FIGURE 6:**
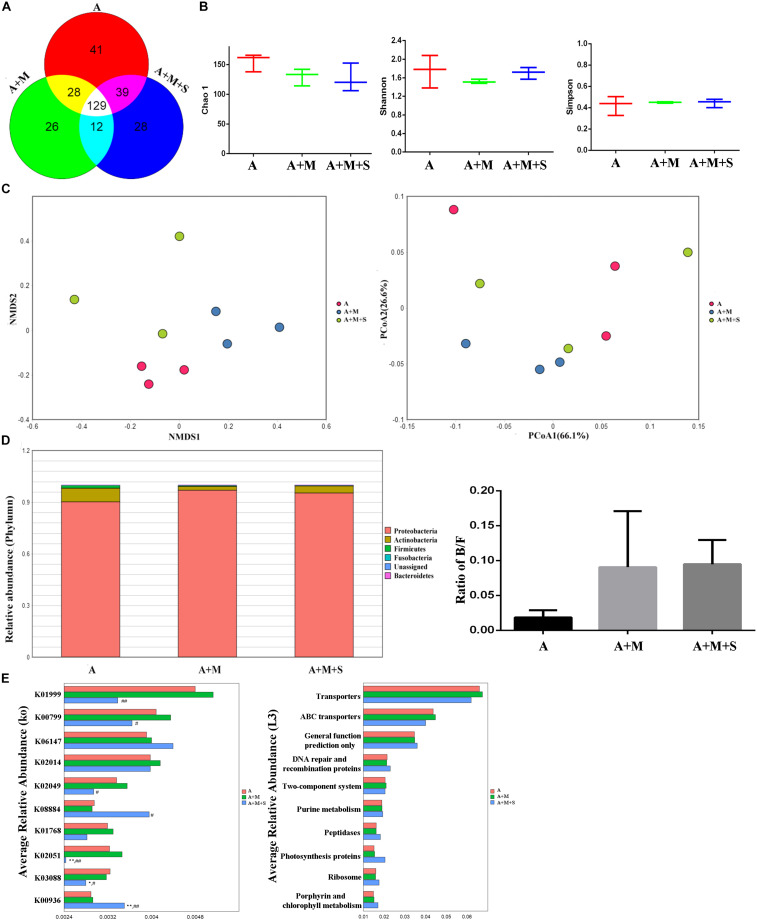
Effect of antibiotic use on the sinomenine treatment of the morphine-dependent gut microbiome. **(A)** Venn diagram depicting the distribution of bacterial taxa (at the OTU level) in the different experimental groups of zebrafish. **(B)** Alpha diversity of the fecal microbiome among the three groups according to the Chao 1, Richness, Shannon, and Simpson indices. **(C)** Beta diversity of the fecal microbiome among the three groups based on principal coordinate analysis (PCoA) and nonmetric multidimensional scaling (NMDS) at the OTU level based on the Bray–Curtis similarity algorithms. **(D)** Comparison of species composition among the fecal microbiota of the A, A+M, and A+M+S groups at the phylum level. **(E)** Prediction of changes in KEGG pathways using PICRUST analysis. *P < 0.05, **P < 0.01 vs the A group; ^#^*P* < 0.05, ^##^*P* < 0.01 vs the A+M group. A, antibiotic group; A+M, antibiotic+morphine group; A+M+S, antibiotic+morphine+sinomenine group.

As shown by the Venn diagram (Figure 6A), a total of 303 OTUs were identified among all the samples, 129 of which were shared by the three groups. A total of 41 OTUs were unique to the A group, 26 to the A+M group and 28 to the A+M+S group. After treatment with antibiotics, there were no significant differences in the community richness and diversity of intestinal microbiota in any of the zebrafish groups (Figure 6B). Distance matrices (beta diversity) between samples were generated based on Bray–Curtis similarity algorithms at the OTU level, and are reported according to PCoA and NMDS (Figure 6C). The PCoA and NMDS results show smaller differences in microbial profiles among the three groups (A, A+M, and A+M+S).

As shown in Figure 6D, the zebrafish GM is mainly divided into five phyla: Proteobacteria, Fusobacteria, Firmicutes, Bacteroidetes, and Actinobacteria. Interestingly, there were no significant differences in the composition of intestinal microflora of zebrafish in any of the groups after antibiotic treatment, indicating that the use of antibiotics caused a disorder in the intestinal microbiota of zebrafish.

As shown in Figure 6E, after antibiotic administration, morphine treatment did not change the abundances of KEGG pathways in the zebrafish GM. However, sinomenine treatment did significantly reduce expression of the following genes: branched-chain amino acid transport system substrate-binding protein (K01999); glutathione S-transferase (K00799); NitT/TauT family transport system ATP-binding protein (K02049); NitT/TauT family transport system substrate-binding protein (K02051); and RNA polymerase (K03088). Furthermore, it increased the expression of genes encoding: serine/threonine protein kinase (K0884); and sensor histidine kinase PdtaS (K00936). At level 3 in the KEGG analysis, there were no differences in the pathway abundances of intestinal microbiota in any of the zebrafish groups.

### Antibiotic Use Results in the Inability of Sinomenine to Inhibit MGBA-Relative Gene Alterations in Zebrafish

**FIGURE 7:**
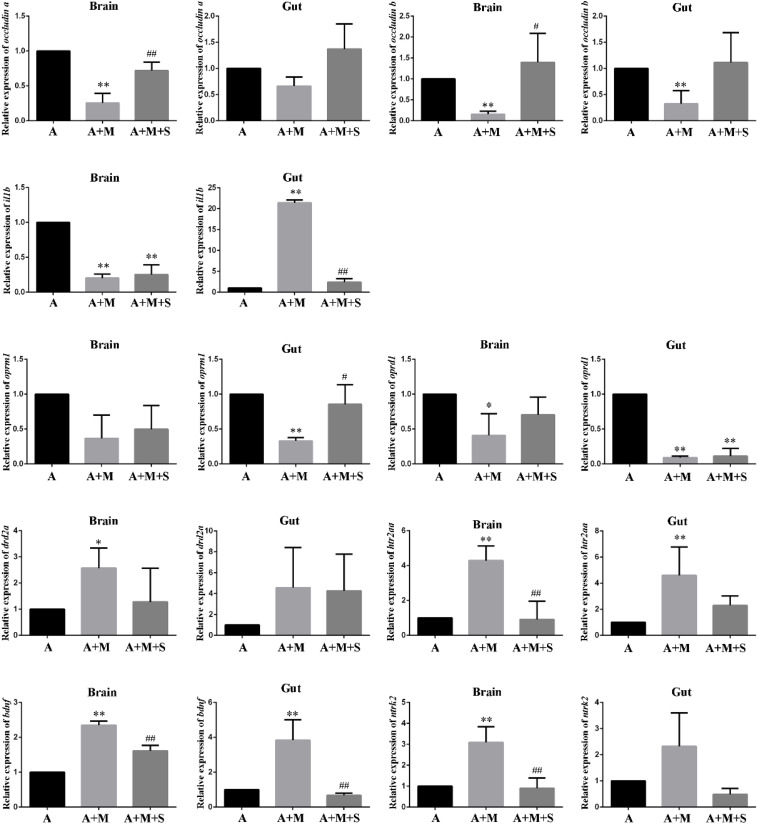
qPCR analysis of *occludin a*, *occludin b*, *il1b*, *oprm1*, *oprd1*, *drd2a*, *htr2aa*, *bdnf*, and *ntrk2* expression in zebrafish. (*n* = 3). *P < 0.05, **P < 0.01 vs the A group; ^#^*P* < 0.05, ^##^*P* < 0.01 vs the A+M group. A, antibiotic group; A+M, antibiotic+morphine group; A+M+S, antibiotic+morphine+sinomenine group.

As shown in Figure 7, compared with the A group, the expression levels of *occludin a* mRNA in the zebrafish brain and *occludin b* mRNA in the zebrafish brain and intestine were significantly reduced after morphine treatment. Only part of the tight junction protein expression returned to normal after sinomenine treatment.

Compared with the A group, after morphine treatment, the expression of *il1b* mRNA in the zebrafish brain decreased significantly, whereas it increased significantly in the zebrafish intestine. However, treatment with sinomenine was only able to reverse the increase in *il1b* mRNA expression caused by morphine in the zebrafish intestine.

Compared with the A group, the expression levels of *oprm1* mRNA in the zebrafish brain and *oprd1* mRNA in the zebrafish brain and intestine decreased significantly in A+M group. The sinomenine intervention could only locally up regulate the expression of *oprd1* mRNA in the zebrafish brain.

Compared with the A group, the expression levels of *drd2a* mRNA in the zebrafish brain and the expression of *htr2aa* mRNA in the zebrafish brain and intestine in the A+M group were significantly increased. Furthermore, compared with the A+M group, there were no significant changes in the expression levels of *drd2a* and *htr2aa* mRNAs in zebrafish from the A+M+S group.

Compared with the A group, the expression of *bdnf* mRNA in the zebrafish brain and gut, and the expression of *ntrk2* mRNA in the zebrafish brain significantly increased after morphine treatment; the sinomenine intervention inhibited these changes.

## Discussion

Morphine has excellent sedative and analgesic effects and is a first-line drug for the clinical treatment of pain. However, its long-term use can lead to addiction and can easily cause symptoms such as nausea, constipation, abdominal pain, and diarrhea (Daoust et al., 2019). Previous research on morphine dependence has focused on the CNS. In recent years, based on research into the interaction between the intestinal microbiota and the brain, researchers have gradually realized these comprise an organic whole. As such, research on brain diseases cannot be limited to the CNS, as normal peripheral function is closely related to the normal operation of the CNS.

Drug dependence includes physical dependence and psychological dependence. Psychological dependence refers to the body’s psychological demand for a substance after long-term use of a certain drug (Hammami-Abrand et al., 2016). In basic research, the CPP is often used to study the potential and characteristics of psychological drug dependence. Previous studies have shown that when the CPP test is performed on humans, test subjects show a preference for the side with the drug after being given the addictive substance, similar to non-human animals. This suggests that the subjective response to the drug is closely related to the body’s positional preference (Childs and de Wit, 2009, 2013). These findings show that by using non-human animals to perform CPP experiments, human drug-dependent behavior can be simulated. Therefore, this experiment used the CPP test to establish a zebrafish morphine dependence model for research.

Before the experiment, each zebrafish was tested for its natural position preference, which is consistent with the previous experimental studies (Zhu et al., 2017b). Most zebrafish showed a preference for the black box, so the black box was selected as the preference box and the white box was selected as the drug-pair box. After 5 days of CPP training, the residence time of the zebrafish in the M group increased significantly in the drug-pair box, indicating that the zebrafish morphine dependence model had been successfully established. Compared with the M group, the residence time of the M+S group zebrafish in the white box was significantly reduced, suggesting that the sinomenine intervention inhibited the morphine-induced CPP.

An increasing number of studies have shown that changes in GM affect bodily behavior. Compared with SPF mice, GF mice exhibit anxiolytic-like behaviors in elevated cross-maze experiments (Neufeld et al., 2011); However, unlike SPF mice, intraperitoneal injection of LPS does not induce depression-like behaviors in GF mice (Campos et al., 2016). Hoban et al. (2016) found that after chronic depletion of intestinal microbiota in SD rats by antibiotics, rats show deficits in spatial memory in water maze tests and depression-like behavior in forced swimming experiments. Not only the depletion of the intestinal microbiota but also the artificial supplementation of beneficial bacteria can affect the bodily behavior. Clinical studies have found that oral supplementation of probiotics to patients with depression is beneficial in reducing their depressive symptoms (Akkasheh et al., 2016). The administration of *Lactobacillus plantarum* PS128 to GF mice significantly increased the total distance traveled by the mice in field tests and reduced the time spent in the elevated maze test (Liu et al., 2016). These results prove that the intestinal microbiota can induce changes in the bodily mood and behavior.

Consistent with previous results, compared with the A group, the residence time in the drug-pair box of the A+M group increased significantly (*P* < 0.05), suggesting that after the use of antibiotics, morphine-dependent zebrafish established a CPP successfully. Interestingly, compared with the A+M group, there was no significant change in the residence time of zebrafish from the A+M+S group in the white box (*P* ≥ 0.05). This result is inconsistent with the previous CPP results, which may be due to the use of antibiotics on the zebrafish. This suggests that antibiotic administration may influence the influence of sinomenine on morphine dependence.

In this study, 16S rRNA sequencing was used to analyze the composition of the intestinal microbiota of zebrafish and the effects of the sinomenine intervention. The results showed that morphine treatment significantly decreased the community richness and diversity of the zebrafish intestinal microbiota. Specifically, the Chao 1, Simpson, and Shannon indices were significantly reduced; sinomenine treatment was able to reture these indices to normal. According to the beta diversity analysis, the species composition of the zebrafish intestinal microbiota in each group was different, with the samples in group C and group M being the most distant. This indicates that the composition difference between these two groups was the largest. Our results suggest that morphine addiction changed the characteristics of the zebrafish GM and that sinomenine intervention was able to inhibit these changes.

The results of alpha diversity analysis showed that there were no significant differences in the community richness and diversity of the zebrafish intestinal microbiota in the three groups receiving antibiotic treatment. Furthermore, the results of PCoA and NMDS showed that the distance between the groups decreased after antibiotic treatment, indicating that the differences in zebrafish GM from each group became smaller. This result is consistent with the result from the alpha diversity analysis, suggesting that the effect of antibiotic administration on the zebrafish intestinal microbiota is greater than that of morphine dependence or sinomenine treatment.

Numerous studies have shown that abnormalities in the CNS results in changes to the intestinal microbiota. Clinical research has found that compared with healthy people, the GM composition and proportion in patients with active depression and in patients with reactive depression change by the same amount; that is, the relative abundance of the Firmicutes phylum is reduced and those of the Proteobacteria, Bacteroidetes, and Actinobacteria phyla are significantly increased (Zheng et al., 2016). Similar to depressive patients, an increase in the relative abundance of the Bacteroidetes and Actinobacteria phyla was reported in patients with autism (Finegold et al., 2010). Based on these characteristic results, we analyzed the composition of the zebrafish intestinal microflora. Compared with the C group, the relative abundance of Fusobacteria in the zebrafish intestinal microbiota of the M group was significantly increased, while the relative abundance of Actinobacteria was significantly reduced. Meanwhile, the relative abundance of Proteobacteria did not change significantly, but it did show a downward trend. The B/F value is often used as an indicator of the floral susceptibility in a diseased state. Significant increases in B/F values were found in cocaine users, autistic patients, breast cancer patients, patients with fatty hepatitis, and patients with depression (Volpe et al., 2014; Jiang et al., 2015; Sobhonslidsuk et al., 2018; Xue et al., 2018). In this study, after administration of morphine, the B/F value in the zebrafish intestinal microbiota was significantly up-regulated; moreover, the change could be reversed by sinomenine intervention. After antibiotic administration, there were no significant differences in the B/F values in any of the groups, which is consistent with the characteristics of the zebrafish intestinal microbiota after antibiotic use. This result suggests that the use of antibiotics on zebrafish did not deplete the microorganisms in the zebrafish intestine. Instead it changed the composition and proportion of the intestinal microflora, causing intestinal microbiota disturbances. This may be the basis for the change resulting from morphine dependence and treatment in zebrafish.

Based on the above analysis of the characteristics of intestinal microflora in zebrafish, PICRUSt was used to analyze the intestinal microfloral function of zebrafish, in order to relate the composition to the function of the intestinal microflora. The results showed that morphine treatment significantly decreased expression of following genes: serine/threonine protein kinase (K08884); putative transposase (K07496); two-component system (K00936); putative ABC transport system permease protein (K02004); and phosphate transport system substrate-binding protein (K02040); Furthermore, it increased the gene expression of: iron complex outer-membrane receptor protein (K02014), glutathione S-transferase (K00799) and RNA polymerase (K03088). At level 3 of the KEGG analysis, the abundances of the following metabolic pathways were significantly altered: transporter; ABC transporters; DNA repair and recombination proteins; photosynthesis proteins; ribosome; two-component system; peptidases and porphyrin; and chlorophyll metabolism. Some of these pathways have been shown to relate closely to the formation of a drug addiction (Shafer et al., 1990; Carvalho et al., 2001; Chaves et al., 2014; Scofield et al., 2016). Consistent with the analysis of the intestinal floral composition, the expression levels of the majority of genes involved in these pathways were significantly reversed after sinomenine treatment, suggesting that sinomenine may play a therapeutic role in morphine dependence by regulating the metabolism of the intestinal microbiota. The changes in the metabolic pathways among the three groups treated with antibiotics were not significant, again suggesting that disorder of the intestinal microbiota might affect drug efficacy by affecting its function.

The MGBA primarily operates via the endocrine, metabolic, immune, and neural pathways. Based on the MGBA, this study focused on the immune pathway and on the neural pathways, analyzed MGBA-related genes, and studied mechanisms related to the intestinal microbiota, to morphine dependence and to sinomenine.

The tight junction protein plays a regulatory role in cell permeability, mainly via scaffold protein (ZO-1) and transmembrane protein (occludin). Methamphetamine can destroy the blood-brain barrier, leading to increased blood-brain barrier permeability and to a reduced expression of tight junction protein, which then induces neuroinflammation (Martins et al., 2011; Northrop and Yamamoto, 2015; Turowski and Kenny, 2015). Furthermore, this drug can also reduce the level of ZO-1 protein in the rat colon and change colon permeability (Persons et al., 2018). In this study, compared with the C group, the expression levels of *occludin a* and *occludin b* mRNAs in the zebrafish brain and intestine were significantly reduced in the M group. Compared with the M group, the expression levels of *occludin a* and *occludin b* mRNAs in the zebrafish brain and intestine were significantly increased in the M+S group.

Exposing GF mice to the intestinal microbiota from SPF mice reduces blood-brain barrier permeability and increases expression of tight junction proteins in the mice (Braniste et al., 2014). Similarly, disorders of the intestinal microbiota can cause uncontrolled proliferation of pathogenic bacteria in the intestine, produce a large number of toxins, and reduce the expression of intestinal tight junction protein, eventually causing damage to the intestinal mucosal barrier (Zyrek et al., 2007). After antibiotic treatment, the expression levels of *occludin a* and *occludin b* mRNAs in the brain and intestine of zebrafish from the A+M group were down-regulated. The intervention with sinomenine was unable to completely antagonize the changes in tight junction protein expression caused by morphine in the zebrafish. Tight junction proteins regulate the diffusion of microorganisms, toxins, and various other molecules from the lumen to the lamina propria and systemic circulatory system. Our results indicate that the intestinal flora may affect the expression of tight junction protein, change the body’s barrier function, and cause changes in various factors in the body, which then influence the formation and the treatment of morphine dependence.

Long-term use of opioids seriously damages the immune system and increases the risk of opportunistic infections (Roy and Loh, 1996; Nath et al., 2002; Quaglio et al., 2002; Roy et al., 2006). However, studies have shown that exogenous substances such as morphine can induce inflammation and increase the expression of IL-1β in vivo (Wen et al., 2011). IL-1β is commonly used as an indicator to evaluate immune capacity. Studies have found that the expression of IL-1β in the serum of morphine-dependent rats is significantly reduced (Wang et al., 2005). In both the normal and disordered intestinal microbiota conditions, after intraperitoneal injection of morphine, the expression of *il1b* mRNA in the brain of zebrafish was significantly reduced, while the expression of *il1b* mRNA in zebrafish intestine was significantly increased. Thus, different changes in the expression of inflammatory factors in the zebrafish brain and intestine were caused by morphine dependence. This result may be related to the method of morphine administration and to the immune response of tissues and organs; as such, an understanding of the detailed reasons would require further study. A healthy intestinal microbiota has a positive effect on immune regulation. Administration of *fructooligosaccharide* and *galactooligosaccharide* (both probiotics) can reduce the release of inflammatory factors in the brain of mice caused by chronic stress (Burokas et al., 2017). Under normal conditions, sinomenine treatment can antagonize changes in the expression of *il1b* mRNA caused by morphine dependence. When the intestinal microbiota is disturbed, sinomenine can no longer completely antagonize changes in *il1b* mRNA expression. This suggests that the therapeutic effect of sinomenine on morphine may be related to the immune system and that the intestinal microbiota may influence the therapeutic effect of sinomenine on morphine via the immune pathway.

Studies have shown that morphine addiction can down-regulate the expression of OprM and OprD in rats and zebrafish. Moreover, OprM and OprD agonists can induce CPP behavior in rats (Tempel et al., 1988; Childs and de Wit, 2009). However, opioid receptors are not only present in the CNS, but are also abundantly expressed in the periphery. The gastrointestinal tract digestive system is jointly regulated by the CNS and the peripheral enteric nervous system (ENS). Opioids can exert effects on the gastrointestinal tract via the CNS and the ENS. Many studies have focused on the mechanisms underlying gastrointestinal diseases caused by opioid receptors and opioids. However, there are few studies on the effect of morphine dependence on the expression of opioid receptors in zebrafish. Consistent with previous studies, the same changes were observed in the brain and intestine of zebrafish from the M and A+M groups; that is, the expression levels of *oprm1* and *oprd1* mRNAs were significantly down-regulated. Compared with the M group, the expression levels of *oprm1* and *oprd1* mRNAs in zebrafish from the M+S group were significantly reduced. Compared with the A+M group, the expression level of *oprm1* mRNA in the zebrafish intestine significantly increased in the A+M+S group. This result is consistent with the results of the CPP teat, suggesting that the intestinal microbiota may affect the therapeutic effect of sinomenine on morphine via the opioid receptor pathway.

Under normal physiological conditions, the intestinal microbiota maintains a dynamic balance, which can protect and maintain the health of the body. However, a change of the intestinal microbiota can lead to a disorder in the signal perception, transmission and response of intestinal epithelial cells to intestinal bacteria, can damage the intestinal mucosa, and can change levels of inflammatory factors and neurotransmitters, eventually affecting brain function (Goto and Kiyono, 2012). Similar to opioid receptors, most neurotransmitters are highly expressed in the brain and gut. Since the brain is the focus of drug addiction and the intestine is an important component of the MGBA, great attention has been paid to the neurotransmitters in these two major tissues. A variety of neurotransmitter receptors in the brain are involved in learning, memory, behavioral, and emotional regulation, including, for example, the dopamine (DA) and 5-HT receptors (Bergquist et al., 2002; Cai et al., 2010). Studies have shown that dopamine D2 receptor (Drd2)-deficient mice do not show a CPP effect after morphine administration. In addition, Drd2 antagonists can effectively reduce morphine tolerance and drug-seeking behavior in rats (Ozdemir et al., 2013). Significant changes in the expression of DA transmitters and Drd2 in the stomach and duodenum of morphine-dependent CPP rats have also been reported (Cai et al., 2010). The activation of 5-HT receptors and the release of neurotransmitters can affect the process of drug addiction and the conduction of nerve excitation (Weitemier and Murphy, 2009; Gomez-Milanes et al., 2012). Morphine can also act on opioid receptors in GABAergic neurons, thereby affecting the release of 5-HT and DA (Tao and Auerbach, 2002). In this study, whether or not antibiotics were used, the expression levels of *drd2a* and *htr2aa* mRNAs in the zebrafish brain and intestine were significantly upregulated after exposure to morphine. Similar to the results for opioid receptors, sinomenine intervention effectively antagonize the up-regulation of neurotransmitter expression in the zebrafish caused by morphine. After antibiotic treatment, sinomenine is only able to reduce the expression of *htr2aa* mRNA in the zebrafish brain caused by morphine. These results suggest that neurotransmitters play an important role in morphine addiction and that the therapeutic effect of sinomenine on morphine dependence cannot be separated from good intestinal conditions.

Numerous studies have demonstrated that BDNF and its receptor Ntrk2 are involved in the regulation of brain function and behavior. Recent studies have found that BDNF is abundantly expressed in the enteric plexus system, and that BDNF and Ntrk2 may exert an influence via the ENS (Acosta et al., 2015). It has been shown that morphine exposure causes a significant up-regulation of BDNF expression in the brain of rats and zebrafish (Liang et al., 2011; Jimenez-Gonzalez et al., 2016), such that, during the morphine withdrawal phase, both BDNF and Ntrk2 expression increase in the brain. In the current study, morphine treatment caused a significant increase in the expression levels of *bdnf* and *ntrk2* mRNAs in the brain and intestine of zebrafish. After treatment with sinomenine, the expression levels of *bdnf* and *ntrk2* mRNAs were effectively down-regulated. After antibiotic treatment, the expression levels of *bdnf* and *ntrk2* mRNAs in zebrafish were similar to the above results. From this, it can be concluded that the treatment effect of sinomenine on morphine dependence may be related to the BDNF/Ntrk2 signaling pathway.

## Conclusion

In summary, our research demonstrates the following: that morphine dependence causes changes in the zebrafish GM; that antibiotic-driven changes in the zebrafish intestinal microbiota also affect the formation and treatment of morphine dependence; and that the interaction between intestinal microbiota and morphine dependence is accompanied by the expression of MGBA-related genes. In addition, we also found that the antibiotic-driven disturbance in zebrafish intestinal microbiota may influence the therapeutic efficacy of sinomenine on morphine via the MGBA; the mechanism of action underlying this needs to be further studied. Our findings provide a novel framework for understanding the mechanism of morphine addiction via the MGBA and may provide new therapeutic strategies for the treatment of drug addiction.

## Data Availability Statement

The Illumina reads for the WGS and RNA-seq experiments have been uploaded to the NCBI Short Read Archive (SRA) under Biosample accession number SRR10915998-6006.

## Ethics Statement

The animal study was reviewed and approved by the Experimental Animal Ethics Committee of Southern Medical University.

## Author Contributions

ZC and MZ conceived and designed the study. ZC, ZY, and LJ performed the experiments. ZC, OJ, CZ, and XS prepared the samples and the sequencing libraries. ZC and MZ, drafted the manuscript. LJ, LC, and ZQ critically reviewed the manuscript.

## Conflict of Interest

The authors declare that the research was conducted in the absence of any commercial or financial relationships that could be construed as a potential conflict of interest.

## Footnotes

1 https://github.com/OpenGene/fastp

2 https://github.com/marcelm/cutadapt/

3 https://www.arb-silva.de/
